# A Novel Banana Mutant “*RF 1*” (*Musa* spp. ABB, Pisang Awak Subgroup) for Improved Agronomic Traits and Enhanced Cold Tolerance and Disease Resistance

**DOI:** 10.3389/fpls.2021.730718

**Published:** 2021-09-23

**Authors:** Xiaoyi Wang, Anbang Wang, Yujia Li, Yi Xu, Qing Wei, Jiashui Wang, Fei Lin, Deyong Gong, Fei Liu, Yanting Wang, Liangcai Peng, Jingyang Li

**Affiliations:** ^1^Hainan Banana Healthy Seedling Propagation Engineering Research Center, Haikou Experimental Station, Chinese Academy of Tropical Agricultural Sciences, Haikou, China; ^2^The Fruit Tree Research Center, Institute of Subtropical Crops, Guizhou Academy of Agricultural Sciences, Xinyi, China; ^3^Biomass and Bioenergy Research Centre, College of Plant Science and Technology, Huazhong Agricultural University, Wuhan, China

**Keywords:** “*ReFen 1*” mutant, Pisang Awak (ABB), ethyl methanesulfonate (EMS)-mutagenesis, semi-dwarfing, agronomic traits, cold tolerance, sigatoka disease resistance, banana breeding

## Abstract

Banana is a major fruit crop grown in tropical and subtropical regions worldwide. Among cultivars, “FenJiao, FJ” (*Musa* spp. ABB, Pisang Awak subgroup) is a popular variety of bananas, due to its better sugar-acid blend and relatively small fruit shape. However, because the traditional FJ variety grows relatively high in height, it is vulnerable to lodging and unsuitable for harvesting. In this study, we sought desirable banana mutants by carrying out ethyl methanesulfonate (EMS) mutagenesis with the FJ cultivar. After the FJ shoot tips had been treated with 0.8% (v/v) EMS for 4 h, we obtained a stably inherited mutant, here called “*ReFen 1*” (*RF1*), and also observed a semi-dwarfing phenotype. Compared with the wild type (FJ), this *RF1* mutant featured consistently improved agronomic traits during 5-year field experiments conducted in three distinct locations in China. Notably, the *RF1* plants showed significantly enhanced cold tolerance and Sigatoka disease resistance, mainly due to a substantially increased soluble content of sugar and greater starch accumulation along with reduced cellulose deposition. Therefore, this study not only demonstrated how a powerful genetic strategy can be used in fruit crop breeding but also provided insight into the identification of novel genes for agronomic trait improvement in bananas and beyond.

## Introduction

Banana is one of the most important fruits and cash crops in tropical and subtropical regions around the world. Currently, bananas are grown in more than 130 countries, and global banana production reached ca. 125 million tons in 2017 (FAOSTAT, 2020), providing both food and income for ca. 400 million people worldwide. China is a major producer and consumer of bananas, whose harvested area and total output were 380 thousand hm^2^ and 11 million tons, respectively, in 2017. In particular, as one of the major banana-growing areas (Perrier et al., 2011), South China has domestically cultivated the bananas for more than 3,000 years now (Ge et al., 2005). However, the number of commercially grown banana varieties is relatively limited, with “Cavendish” being the dominant cultivar (de Deus et al., 2018), it accounts for more than 85% of all banana production in China.

“FenJiao, FJ” (*M. paradisiaca* Pisang Awak subgroup ABB), like Cavendish (*M. acuminate* Colla, group AAA), belongs to the *Musa* genus, a large herb of the family Musaceae (Cheesman, 1947). FJ is also known as a ‘milk banana’ in Hainan, China due to its sweet taste; it is also a desirable banana for cooking because it does not brown when cut and remains firm when cooked (Chong et al., 2011; Bi et al., 2017). Given its abiotic stress-resistant properties, FJ has a stable market price (Naknaen et al., 2016) and can be grown in some subtropical regions of China (Hu et al., 2015), and its fruit can ripe quickly, distinguished by a high respiration rate and substantial ethylene production (Zhu et al., 2018b). Furthermore, FJ enjoys the dual advantages of good taste and high nutrient content (Wang et al., 2019), and its consumption is assumed to be associated with a strong reduction in the risk of colorectal cancer (Deneo-Pellegrini et al., 1996), which harbors antifungal (Ranasinghe et al., 2002) and antibacterial effects (Ono et al., 1998). However, the local FJ varieties in Hainan have disadvantages to some degree, such as higher plants, a longer growth cycle, and being vulnerable to Sigatoka disease (Huang et al., 2010; Pattison et al., 2014; Guo et al., 2015; Rames et al., 2018; Pegg et al., 2019; Shao et al., 2020), which limits the widespread cultivation of this banana plant. The traditional breeding method of genetic crossing has been applied with difficulty to dessert banana varieties, given the genetic characteristics of banana in general: parthenocarpic, polyploidy, irregular meiotic behavior, low fertility, and seed viability, among others (Heslop-Harrison and Schwarzacher, 2007; Jeridi et al., 2012; Arinaitwe et al., 2019; Batte et al., 2019). However, somatic cell mutations do not offer an effective approach for screening new varieties of banana (Bhagwat and Duncan, 1998; Karmarkar et al., 2001; Reyes-Borja et al., 2007; Uma et al., 2012; Amah et al., 2019). A mutation is, in principle, based on sudden heritable changes in the genetic material of an organism under abnormal genetic separation or recombination, such as spontaneous and induced mutations (Oladosu et al., 2015). As the incidence of spontaneous mutation is very low, physical and chemical mutagenesis agents play a pivotal role in mutagenesis (Griggs et al., 2013). In the past few decades, mutation breeding has produced thousands of new crop varieties (Sima et al., 2017). In this respect, ethyl methanesulfonate (EMS) is one of the most effective, reliable, and powerful mutagens; treating plants with EMS could destroy their nuclear DNA and randomly induce new mutations in the process of DNA repair (Greene et al., 2003; Jain, 2010). Creating new germplasm through EMS has been implemented for many crops, such as barley (Caldwell et al., 2004), rapeseed (Lee et al., 2018), potato (Moon et al., 2018), rice (Serrat et al., 2014), wheat (Wang et al., 2018), maize (Zhang et al., 2020a), and Chinese cabbage (Lu et al., 2016). Notably, Jankowicz-Cieslak et al. (2012) treated the meristem of banana stem tips with EMS and obtained high-density GC-AT base pair transition mutations, which demonstrated the high efficiency of EMS in banana germplasm innovation. Here, we used the local FJ variety in Hainan as parent material for EMS mutagenesis. Specifically, we established a technical platform suitable for asexual reproduction and generated mutant populations of FJ, leading us to obtain a semi-dwarf mutant with stable genetic traits through screening. We termed this mutant *ReFen 1 (RF1)* and examined its characteristics with respect to low temperature resistance, Sigatoka disease resistance, and suitability to marginal soils. Besides finding markedly improved morphological and agronomic characteristics of *RF1* mutant when grown across different ecological experimental sites, this study also provides insight into new germplasm creation via chemical mutagenesis.

## Materials and Methods

### Establishment of EMS Mutation Technology

*In vitro* plantlets of local FJ (Xinglongnaijiao, Pisang Awak subgroup) were subjected to mutagenesis by using EMS (Sigma-Aldrich, Burlington, MA, USA). Proliferating shoot clusters were maintained *in vitro* through aseptic shoot tip cultures in Murashige and Skoog (MS) basal media (Murashige and Skoog, 1962) supplemented with 3.0 mg/L 6-benzylaminopurine (BAP), 1.0 mg/L adenine, 3.0 g/L agarose, and 30 g/L sucrose, at pH 5.8. Proliferating shoot clusters received an EMS solution treatment by cutting a single isolated shoot tip, using a scalpel blade. These single shoots were incubated with EMS solution on a constant temperature (27°C) rotary shaker at 120 rpm. A series of EMS solutions with different percentages of EMS was prepared using sterilized water plus 1% Tween-80, with each solution sterilized by filtration.

We expected a moderate-to-high-mutation density in the genome of progeny obtained from FJ banana mutagenesis, yet the lethality of M1 plants under a high dose of mutagen precluded producing many plantlets in a single trial. Therefore, we set up a crossed factorial experiment using a series of mutagen concentrations and immersion time gradient tests. Shoot tips were incubated for 4, 8, 16, and 24 h in 0.4, 0.8, 1.2, and 1.6% concentrations of mutagen, for a total of 16 treatment combinations. Each was inoculated with about 10 shoot tips in an Erlenmeyer flask containing 40 ml of prepared EMS mutagen solution. After applying the treatment, shoot tips were rinsed three times in distilled water and then placed into multiplication media (as described above). Mutagenized shoot tips were transferred into fresh multiplication media every 25–60 days, depending on the differentiation of shoot tips observed. After completing six such cycles, the samples were transferred into rooting media that consisted of MS basal media supplemented with 1.0 g/L IBA (Indole-3-Butytric acid), 3.0 g/L agarose, and 30 g/L sucrose, at pH 5.8. The survival ratio, rate of differentiation, days of differentiation, and mutation probability were recorded during this period. After plantlets were allowed to root for 40 d, they were simultaneously acclimatized in a greenhouse for late use in field trials.

### Field Experiments and Plant Growth

The agronomic trials of FJ mutants were evaluated in multiple agro-ecology sites, namely, Danzhou, Haikou, and Xingyi. At each site, the variety comparison test was carried out in an area of 1,250 m^2^, by using a randomized block trial design with three replicates. The plant spacing for these field trials was 2.5 m between rows and 2 m among plants within a row. The hardened sterile tissue culture seedlings were used as the planting seedlings, while the traditional FJ variety served as the control group. The mutant was evaluated *in situ* from 2014 to 2018.

### Observation of Plant Growth and Development

According to the previous studies (Orjeda, 1998; Amah et al., 2019), key agronomic characteristics, namely, vegetative and yield traits, were separately evaluated at flowering time and at harvest time. The height of the pseudo-stem (HP), the pseudo-stem girth (PG) at 100 cm above the soil surface, and the plant base girth (PBG) were recorded at the time of flowering. Days to flowering (DTF) were recorded as the number of days between planting and the onset of flowering. Days to fruit filling (DTFF) were recorded as the number of days between the flowering and harvesting dates. The planting to harvest days (PTHD) was recorded as the number of days between the dates of planting and harvesting. The date of flowering was recorded upon the emergence of the flag leaf, and the date of harvest was recorded the day the banana fruits were harvested and removed from plants. Bunch weight (BW), number of hands (NH), the total number of fingers (NF), fruit weight (FW), fruit length (FL), and fruit circumference (FC) were recorded at harvest when fruits were full-bodied yet unripe (green). Fruit-related traits were collected from the middle fruit of the third hand of each bunch. At each site, 10 banana plants were randomly selected in each replicate. The harvesting criteria/steps were as follows: harvest 85% of the ripe fruit, weigh the fresh fruit after harvest, record the value of weights, subtract 10% of the fruit axis and other discarded tissues to obtain the net yield, and then derive the average value.

### Cold Resistance Assessment

#### Phenotypic Observations

For three production cycles by field natural identification method, five plants were surveyed for chilling injury at the Xingyi test site when low temperatures occurred in winter. Following the technical specifications for the cold damage assessment of bananas, the damage from chilling was recorded (Meteorological Bureau of Guangxi Zhuang Autonomous Region China, 2013). Under natural conditions, the whole plant is then typically graded for the severity of certain features: the upper part of the leaf blade and the apex of the cigar leaf have turned black and dried, or there are light-to-moderate black filaments visible in the inner layer of banana peel (Low). More than 50% of the leaves are wilted, and one-third of the cigar leaf area is damaged (Intermediate). More than 80% of the leaves are wilted and half of the cigar leaf area suffer from dry blight (High). Above-ground leaves and the cigar leaf all suffer from dry blight, having a weak or lost regenerative capacity, and die as a whole (Very high).

#### Cell Wall Composition Assay

The characteristics of plant cell walls may be an important factor in determining the resistance of a plant to cold (Gusta and Wisniewski, 2013). Accordingly, we tried to study the cold-resistance characteristics of *RF1*, FJ, and Baxijiao (BX; Cavendish subgroup) by analyzing the differences in cell wall composition in rachis and pseudo-stems. The banana plant tissues were dried to a constant weight, ground using a grinder, and passed through a 40-mesh screen. Next, the plant cell wall fractionation method was applied, as described previously (Peng et al., 2000; Wu et al., 2013). Each banana biomass sample (0.3 g) was incubated with 6 ml of potassium phosphate buffer (pH 4.8) in a boiling water bath for 1 h and shaken every 10 min. After centrifugation at 3,000 × *g* for 5 min, the supernatant was collected. Hexoses and pentoses of soluble sugars were separately detected by the colorimetric assay, as described by Li et al. (2014). After successive extractions of soluble sugars, lipids, and starch with a phosphate buffer (pH 7.0), chloroform-methanol (1:1, v/v), and (dimethyl sulfoxide, DMSO)–water (9:1, v/v), the remaining crude cell wall pellets were incubated with 0.5% ammonium oxalate monohydrate (w/v) for 1 h in a boiling water bath, to extract the pectin fraction. The leftover residues were incubated with 4 M KOH (containing 1.0 mg/ml sodium borohydride) at 25°C for 1 h, and their supernatants were then collected as KOH-extractable hemicelluloses fraction after centrifugation at 4,000 × *g*. The remnant non-KOH extractable residues were dissolved with H_2_SO_4_ (67%, v/v) at 25°C, for 1 h, and the hexose of the supernatants was then detected as the cellulose fraction (Li et al., 2021). Total hexoses and pentoses of the KOH-extractable hemicelluloses and total pentoses of the non-KOH extractable fraction were summed to express the hemicellulose content level. Likewise, the total contents of hexoses, pentoses, and uronic acids were summed to derive the pectin content level. A UV-vis spectrometer (V-1100D, Shanghai MAPADA Instruments Co., Ltd. Shanghai, China) was used to detect and quantify the hexoses, pentoses, and uronic acids, as previously described (Cheng et al., 2018). A two-step acid hydrolysis method was applied to determine the lignin content of samples, and this was done according to the Laboratory Analytical Procedure of the National Renewable Energy Laboratory, as previously described (Fan et al., 2017). All the experiments were performed independently three times.

### Sigatoka Resistance Assessment

Data on black Sigatoka disease were recorded over two cycles from the planting date, by using the field natural identification method (Vishnevetsky et al., 2011), the field identification of the trial varieties (*RF1*, FJ, and Baxijiao), the whole year does not use any fungicide. Ten plants were randomly selected to investigate the disease severity of each plant; for this, the youngest leaf with at least 10 necrotic spots at flowering (youngest leaf spotted, YLS), number of standing leaves at flowering (NSL) were recorded, from which the index of non-spotted leaves (INSL) (Orjeda, 1998; Smith et al., 2018) was calculated this way:


$$\begin{array}{l} \text{INSL }=\;\frac{\text{YSL}-1}{\text{NSL}}\;\times \;100 \end{array}$$

### Banana Fruit Quality Assay

Bananas fruits were harvested at the mature green stage. When the fruit was fully ripe (i.e., the color of the peel was bright yellow), the fruit quality was analyzed. To do this, several chemical properties—reducing sugar (RS), total sugar (TS), vitamin C (VC), total titratable acidity (TA), and crude protein (CP)—were determined by following the procedures used in other reported research (Workneh et al., 2012; Tigist et al., 2013; Altemimi et al., 2017; Yap et al., 2017). Briefly, an aliquot of banana juice was obtained using a juice extractor and filtered through gauze. The ensuing clear juice was used for further analyses. Using the method described by Tigist et al. (2013), the RS and TS values of banana juice were measured with a refractometric saccharometer (ATAGO® MASTER-53T). The VC content was measured by the 2, 6-dichlorophenolindophenol method (AOAC, 1990), for which a 10-ml banana juice extract was diluted to 50 ml with 3% met phosphoric acid in a 50-ml volumetric flask. This was then centrifuged at 10,000 × g for 15 min, followed by titration with standard dye to a pink end point for 15 s; ascorbic acid content (%) was calculated according to the titration value, dye factor, dilution factor, and volume of the sample. The TA of banana was quantified as described by Yap et al. (2017), in which diluted banana pulp was titrated with 0.1 mol/L NaOH. After adding each NaOH drop, the Erlenmeyer flask was rotated until the color disappeared, faded, or stayed the same after ~1 min of rotating. TA was calculated in grams of malic and citric acid per 100 ml. CP content was determined using the Kjeldahl method, as described by Zhu et al. (2018a).

The ratio of sugar to acid (RSTA) was calculated as follows:


$$\begin{array}{l} \text{RSTA }=\;\frac{\text{Total sugar }\left(\text{TS}\right)}{\text{Total titratable acidity }\left(\text{TA}\right)} \end{array}$$

Potassium, zinc, and iron concentrations were quantified using the flame photometric method. The ash of each banana sample was digested with nitric acid on a hot plate. Then the samples were aspirated into the flame and measurements were made with an atomic absorption spectrophotometer (Aanalyst401, Spectrometer, Perkin Elmer, Waltham, MA, USA) equipped with different lamps for the different mineral elements.

### Data Collection and Statistical Analysis

The results are expressed as the mean ± SD or median of at least three independent experiments. Differences were considered significant at *p* < 0.05. Differences between treatment combination groups were assessed using a one-way ANOVA with a *post-hoc* Least Significant Difference test (LSD) *t*-test, or a two-way ANOVA followed by an LSD-multiple comparisons test, implemented in Sigma Plot 14.0 (Systat Software, San Jose, CA, USA). The 10 plant traits of *RF1* and FJ were used as input values for a principal component analysis (PCA), to check for similarities and differences in agronomic/physiological traits among the samples, this was performed in Origin 2018 software (Origin Lab Corporation, Northampton, MA, USA).

## Results

### Selection of EMS-Induced Mutant With Local Banana Cultivar (FJ)

We examined whether EMS could induce lethal mutagenesis in banana shoot cultures of the local banana cultivar FJ. First, a chemical mutation culture system suitable for FJ was established for mutant screening. Then the toxicity of EMS was inferred from the survival rate and differentiation time of banana proliferation shoots under different combinations of mutagen concentrations and soaking times. The two-way ANOVA results revealed significant effects of EMS concentration, treatment time, and their interaction on mutagen-induced plant responses (Table 1). A significant reduction in the survival rate was observed with an increasing EMS concentration and longer treatment time (*p* < 0.05; Supplementary Figure 1). Lethal dose 50 (LD_50_) is defined as the EMS concentration and treatment time that results in 50% lethality (Arisha et al., 2015). Generally, LD_50_ is used as a parameter to produce a high mutation frequency (Hohmann et al., 2005). When the mutagen concentration was higher than 0.8% and soaked for more than 4 h, the survival rate of M1 was significantly low, at <50% (Table 1; Supplementary Figure 1). Therefore, the LD_50_ value of EMS mutagen with FJ was estimated to be ca. 0.8% under a 4-h soaking.

**Table 1:** Banana shoot growth and development after treatment with EMS.

**EMS concentration (%) and treatment time (h)**	**Number of shoots**	**Changes in shoots**			**Morphological change (%)**
		**Surviving (%)**	**Differentiation (%)**	**Days of differentiation (days)**	
**Untreated controls (liquid MS media)**					
2	64	100.0a	100.0a	8.2d	0
4	62	100.0a	100.0a	8.3d	0
8	60	100.0a	100.0a	8.2d	0
16	63	99.7a	99.9a	8.4d	0
**Concentration (0.2%)**					
2	62	95.5a	93.8a	13.3cd	1.58
4	60	85.4a	82.4a	15.4c	2.61
8	66	57.2bc	63.1c	18.1c	3.59
16	60	36.1cd	55.8cd	22.0bc	5.02
**Concentration (0.4%)**					
2	65	91.7a	88.7a	14.1cd	3.14
4	66	74.5ab	78.4ab	17.6c	5.70
8	62	41.3c	59.6c	21.5c	6.51
16	62	29.4cd	48.9cd	25.4ab	7.20
**Concentration (0.8%)**					
2	60	80.3a	85.2a	18.7c	5.36
4	61	51.4bc	65.0bc	21.0bc	6.62
8	65	31.7cd	52.4cd	25.4ab	8.75
16	60	23.4d	44.1d	29.1a	8.94
**Concentration (1.0%)**					
2	60	73.3ab	80.6ab	20.6bc	6.20
4	65	45.8cd	67.4bc	25.4b	7.42
8	63	29.5cd	49.7d	28.0a	8.95
16	62	17.4d	40.8d	31.4a	9.60
**Concentration (2.0%)**					
2	66	38.4c	59.3c	22.1bc	6.88
4	62	26.6cd	48.1cd	26.4a	8.46
8	65	16.7d	44.0c	29.3a	9.90
16	60	9.8d	29.4c	35.1a	11.2

*Statistical significance was assessed using two-way ANOVAs. Within columns, differing lowercase letters indicate mean values that are significantly different. EMS, ethyl methanesulfonate; MS, Murashige and Skoog*.

Furthermore, the EMS-induced mutation with FJ showed large variation, especially for the incidence of total morphological change, which varied from 1.58 to 11.2%. For instance, white leaf stripe, Xantha, chlorine, and dwarfism were frequently observed in mutants. These mutants were then planted in fields, and we found that a semi-dwarf mutant, here termed *RF1*, had excellent agronomic traits among all the mutants examined.

### Altered Morphological and Botanical Characteristics in *RF1* Mutant

**Figure 1:**
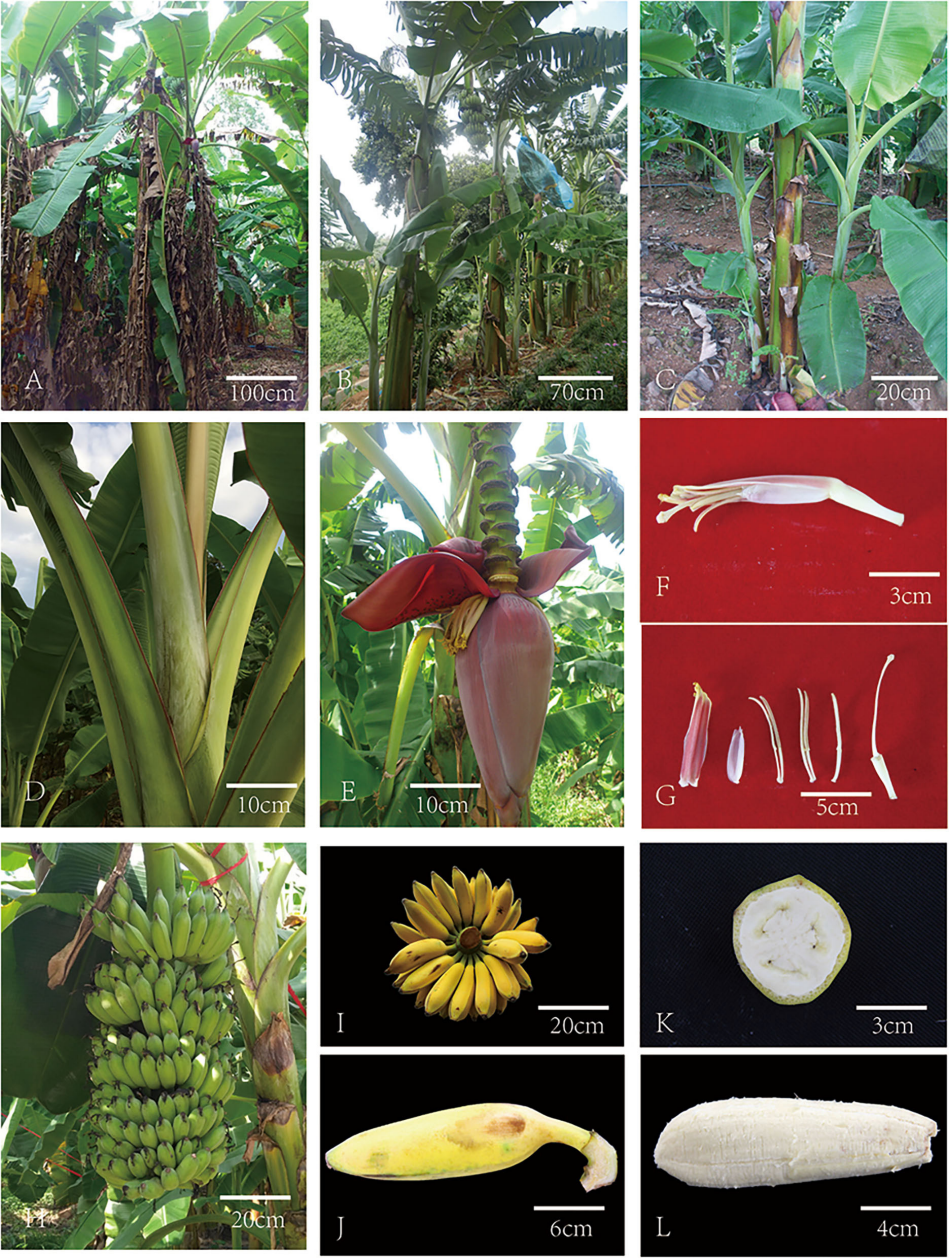
Morphological observations of the ‘*RF1*’ mutant. **(A,B)** FJ (bar = 100 cm) and *RF1* (bar = 70 cm) phenotypes. **(C,D)** *RF1* suckers (bar = 10 cm) and petiole (bar = 20 cm). **(E,F)** RF1 male bud (bar = 10 cm) and male flower (bar = 3 cm). **(G)** *RF1* compound tepal, free tepal, filament ×3, ovary and style (from left to right, respectively). Bar = 5 cm. **(H)** *RF1* bunch on the tree. Bar = 20 cm. **(I)** *RF1* mature fruit. Bar = 20 cm. **(J)** *RF1* fruit shape. Bar = 6 cm. **(K)** Arrangement of ovules of *RF1* fruit. Bar = 3 cm. **(L)** *RF1* fruit pulp. Bar = 4 cm. *RF1, ReFen 1; FJ*, FenJiao.

To characterize the *RF1* mutant, we observed that the pseudo-stem of *RF1* was dwarfed and the pseudo-stem girth became thicker, compared to the FJ cultivar (i.e., the wild type/control). The height of pseudo-stem (HP) was decreased by 32%, while the PG was increased by 25.2% at the *p* ≤ 0.05 level (Table 2). The mature *RF1* mutant had a pseudo-stem height of 3.0–3.2 m, with a 70–80 cm circumference at a height of 100 cm from the ground, and it was covered with a small amount of wax powder (Table 2 and Figures 1A,B). Suckering was close to the parent plant 5–50 cm, to 8–11 suckers, which grew vertically (Figure 1C). The lamina oblong reached 180–220 cm in length and 48–60 cm in width, and the midrib was yellowish-green, suborbicular at the base. Petiole length was 38–60 cm, and leaf sheaths were at green, smooth, and margin incurved by 2–3 cm from both sides of petiole edge (Figure 1D). The bracts lilac was outside, and the inner surface vermillion was 18–30 cm long, deciduous, with 12–20 flowers per bract, double rowed (Figures 1E,F). The compound petals were 5.8–6.5 cm long, yellow-purple, the lateral ones narrow; free petals were 2.5–3.2 cm long, translucent, pale-purple, boat-shaped, with stamens light yellow (Figure 1G). Inflorescence pendulous was 80–120 cm long, and its peduncle and rachis shortly and softly pubescent, arranged in a spiral hand and composed of 5–15 hands, each with 12–24 fruits at 11–22 cm long. Fruit pedicel was at 1.8–2.0 cm long, glabrous (Figures 1H–J). The circumference was 8–15 cm, with slightly curved fingers. The fruit apex was obtuse, and the cross-section of the fruit had five-sided angles (Figure 1K). The peel was green, pericarp at 1.0–1.5 mm, ripening yellow, and the pulp cream in color (Figures 1K,L). Finally, this study confirmed that *RF1* fruits were seedless. Hence, the *RF1* mutant featured markedly altered morphological and botanical properties when compared to the FJ cultivar.

**Table 2:** Major agronomic traits of *RF1* and FJ at multiple field sites.

**Test sites**	**Variety**	**Date**	**DTF (days)**	**DTFF (days)**	**PTHD (days)**	**HP (cm)**	**PBG (cm)**	**PG (cm)**
Danzhou	RF 1	FS	347.9c	72.0a	421.0a	317.5a	108.5a	75.0a
		FR	336.7b	70.3a	409.0a	312.5a	112.5a	79.0a
		SR	329.9a	70.5a	406.0a	306.5a	112.0a	80.0a
	FJ	FS	373.2f	76.2a	452.0b	467.0b	95.0b	59.0b
		FR	359.0d	76.5a	431.0a	455.0b	101.5a	64.0b
		SR	351.9d	76.0a	427.5a	449.5b	100.5a	64.5b
Haikou	RF 1	FS	352.8d	78.5b	432.5a	313.0a	103.5a	77.0a
		FR	336.7b	76.1a	413.0a	305.5a	109.0a	78.0a
		SR	328.9a	71.6a	400.5a	309.0a	111.5a	79.0a
	FJ	FS	382.3g	91.5b	472.0b	461.0b	90.5b	70.0b
		FR	366.3e	90.2b	455.0b	454.5b	92.5b	71.5b
		SR	357.8d	87.7b	444.5b	454.5b	95.0b	72.0b
Xingyi	RF 1	FS	376.2f	89.5b	467.0b	317.0a	104.5a	75.5a
		FR	355.2d	89.2b	443.5b	320.5a	110.0a	75.0a
		SR	348.2c	89.0b	437.0a	315.5a	104.0a	78.0a
	FJ	FS	414.7j	88.5b	502.0b	449.5b	89.5b	66.0b
		FR	395.1i	90.0b	485.0b	446.5b	92.0b	67.5b
		SR	388.6h	88.6b	478.5b	441.0b	92.0b	62.0b

*DTF, days to flowering; DTFF, days to fruit filling; PTHD, planting to harvest days; HP, the height of pseudo-stem; PBG, the plant base girth; PG, the plant girth; FS, the first season; FR, the first ratoon; SR, the second ratoon*.

*Data are the mean ± SD (n = 10). Statistical significance was assessed using one-way ANOVAs. Within columns, differing lowercase letters indicate group values that significantly different (one-way ANOVA, p < 0.05), according to a pairwise multiple comparison procedures (Tukey's test)*.

### Improved *RF1* Agronomic Traits

This study further compared *RF1* and FJ in the Danzhou experimental field from 2014 to 2017 for the testing of two ratoons (Table 3). The *RF1* mutant showed a significantly increased BW by 18.7%, on average, when compared to FJ (*p* ≤ 0.05) in the first season (FS), while the BW was 20.8% higher of *RF1* than FJ in the first ratoon (FR) with a 21.5% increase in BW in the second ratoon (SR) test (*p* ≤ 0.05; Table 3). Accordingly, the *RF1* mutant displayed significantly increased BW, FW, FL, and FC, all at *p* ≤ 0.05 (Table 3). Additionally, the *RF1* had significant advantages in growth cycles, in which DTF, DTFF, and PTHD were shortened by 24, 5, and 27 d at *p* ≤ 0.05, respectively (Table 2).

**Table 3:** Yield assessment of *RF1* and FJ at multiple field sites.

**Test site**	**Variety**	**Harvest date**	**BW (kg)**	**NH**	**NF**	**FW (g)**	**FL (cm)**	**FC (cm)**
Danzhou	*RF1*	FS	24.8c	10.5a	200.5c	123.8a	15.5a	15.6a
		FR	27.3a	12.0a	218.9a	130.8a	15.5a	15.5a
		SR	27.1a	12.0a	220.4a	130.1a	15.7a	15.9a
	FJ	FS	20.9f	10.0b	190.2d	106.1b	13.3b	13.4b
		FR	22.6 e	12.0a	211.4b	112.0b	13.2b	13.4b
		SR	22.3 e	12.0a	216.5a	110.6b	13.6b	13.7b
Haikou	*RF1*	FS	25.2c	11.0a	197.9c	128.0a	15.9a	16.0a
		FR	27.0a	11.0a	195.4c	135.0a	16.0a	16.3a
		SR	26.5b	12.0a	199.9c	134.0a	16.0a	16.2a
	FJ	FS	21.6 e	11.0a	194.8c	110.5b	13.5b	13.9b
		FR	22.0 e	11.0a	193.1c	109.5b	13.9b	14.0b
		SR	21.0f	11.0a	189.6d	110.5b	14.1b	14.3b
Xingyi	*RF1*	FS	22.0 e	11.0a	196.4c	119.5a	15.5a	16.0a
		FR	23.7 d	11.0a	195.6c	125.5a	15.9a	16.0a
		SR	22.9 e	12.0a	201.2c	125.5a	15.9a	16.0a
	FJ	FS	18.5g	11.5a	196.6c	108.5b	13.9b	14.0b
		FR	20.4f	11.0a	200c	113.5b	13.8b	14.0b
		SR	20.7f	11.0a	197.5c	113.5b	13.9b	14.0b

*BW, bunch weight; NH, number of hands; NF, total number of fingers; FW, fruit weight; FL, fruit length; FC, fruit circumference*.

*Data are the median (n = 10). Statistical significance was assessed using one-way ANOVAs. Within columns, differing lowercase letters indicate group values that significantly different (one-way ANOVA, p < 0.05), according to a pairwise multiple comparison procedures (Tukey's test)*.

**Figure 2:**
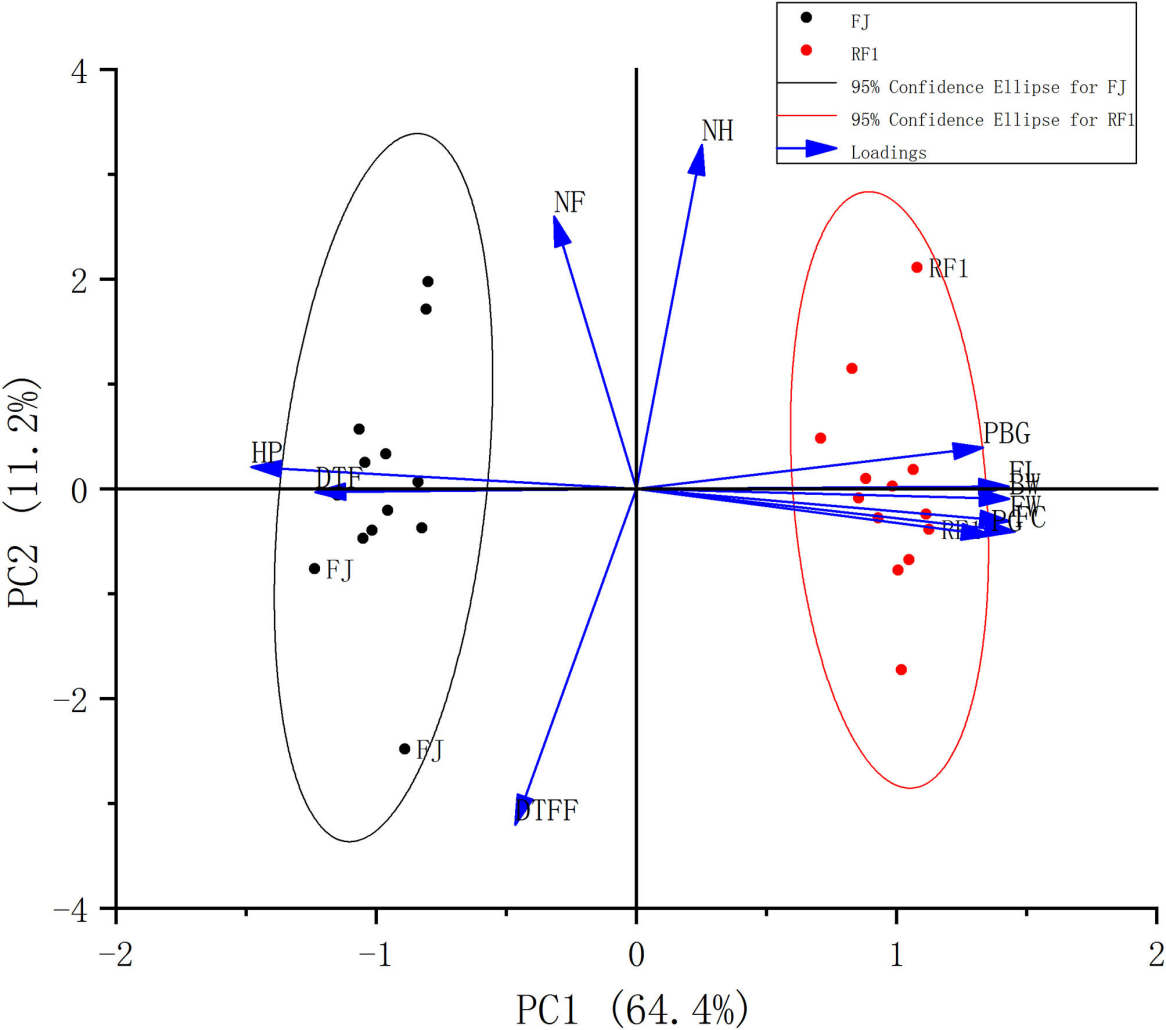
Comparison of 11 agronomic traits between *RF1* and FJ. The distribution of the agronomic traits plotted by principal components analysis (PCA). HP, the height of pseudo-stem; PBG, the plant base girth; PG, the plant girth; DTF, days to flowering; DTFF, days to fruit filling; BW, bunch weight; NH, number of hands; NF, total number of fingers; FW, fruit weight; FL, fruit length; FC, fruit circumference.

Furthermore, a PCA comprehensively evaluated HP, PBG, PG, DTF, DTFF, BW, NH, NF, FW, FL, and FC between the *RF1* mutant and FJ cultivar, to screen the agronomic/physiological traits responsible for the variable discrimination (Tables 2, 3). This showed that two principal components (PCs) together explained 75.6% of the variance in the whole data set (PC1: 64.4%; PC2: 11.2%; Figure 2). The distribution of traits along the two components (PC1 and PC2) is shown in Figure 2. The traits PBG, FL, BW, FW, FC, and PG were associated with *RF1*, whereas HP and DTF were associated with FJ. Meanwhile, the NH, NF, and DTFF traits did not differ between the *RF1* mutant and FJ cultivar. These results suggested that *RF1* offers great advantages in terms of plant growth and development.

In addition, agronomic trials of *RF1* mutant were conducted and evaluated from 2014 to 2018 in multiple agro-ecology sites by Danzhou, Haikou, and Xingyi (Supplementary Figure 2 and Supplementary Table 1). These results indicated that the plant growth of the RF1 mutant was similar between Haikou and Danzhou. By contrast, their DTF, DTFF, and BW of RF1 mutant were all significantly different between Hainan and Xingyi. The altitude of Xingyi exceeds that of Haikou and Danzhou (Supplementary Figure 2), and so the annual mean temperature in Xingyi is 7°-9° lower than that in Hainan (Supplementary Table 1). In Xingyi, plants incurred cold injury when temperatures reached as low as 1°C for 3 d. Banana is suitable for planting at 20–35°C and is severely affected by low temperature, growing poorly at below 10°C. Therefore, based on our results, we speculated that perhaps the low temperature not only caused the extension of the DTF and fruit filling but also led to a reduction in fruit production.

### Altered *RF1* Fruit Nutrient Composition

**Figure 3:**
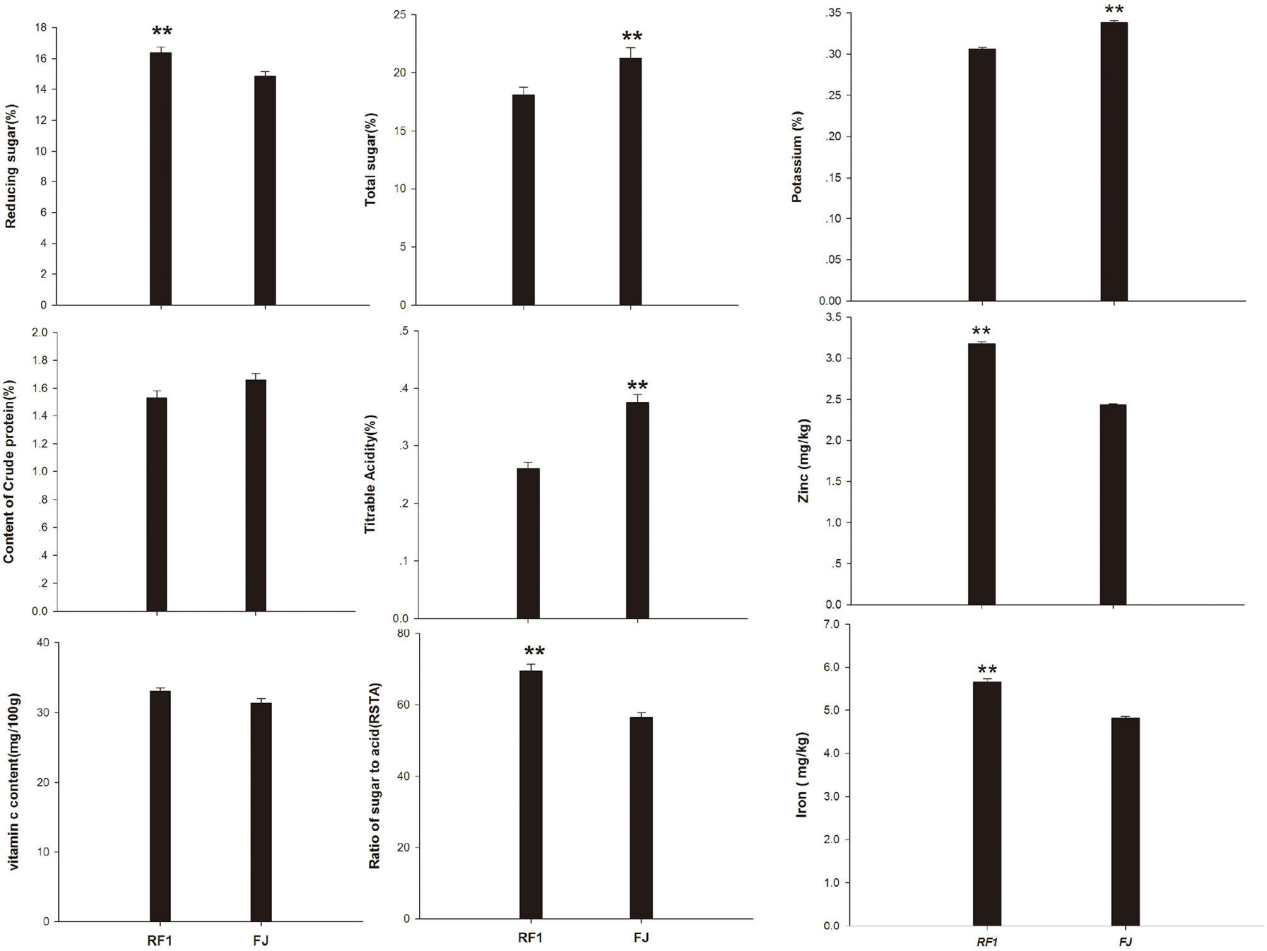
Comparison of fruits nutrients between *RF1* and FJ during fruit ripening stages. Data are the mean ± SD (*n* = 10); One-way ANOVA was used to assess statistical significance (**p < 0.01).

In terms of the fruit quality of *RF1* mutant, this study determined its skin color, shape, size, nutrient content, sweetness, and flavor, etc. (Wyatt et al., 2015). Compared with the FJ cultivar, the *RF1* mutant showed a significantly increased RS content but had reduced TS levels and TA, at *p* ≤ 0.01 (Figure 3). However, the *RF1* mutant contained CP and VC levels similar to those present in the FJ. Given that the RSTA is an important indicator that can affect the taste and quality of fruit (Zhang et al., 2020b), here we uncovered that *RF1* produced bananas of delicate flesh and moderately sweet and sour, whose TS content was 3.17% (lower than FJ); however, TA content of FJ was 0.12% higher than that of *RF1*, therefore, the RSTA of *RF1* was significantly higher (reaching 12.96) than that of FJ at *p* ≤ 0.01. Further, this study found that the *RF1* mutant contained more zinc and iron whereas the FJ had a higher level of potassium content.

### Enhanced *RF1* Tolerance to Cold and Resistance to Disease

**Figure 4:**
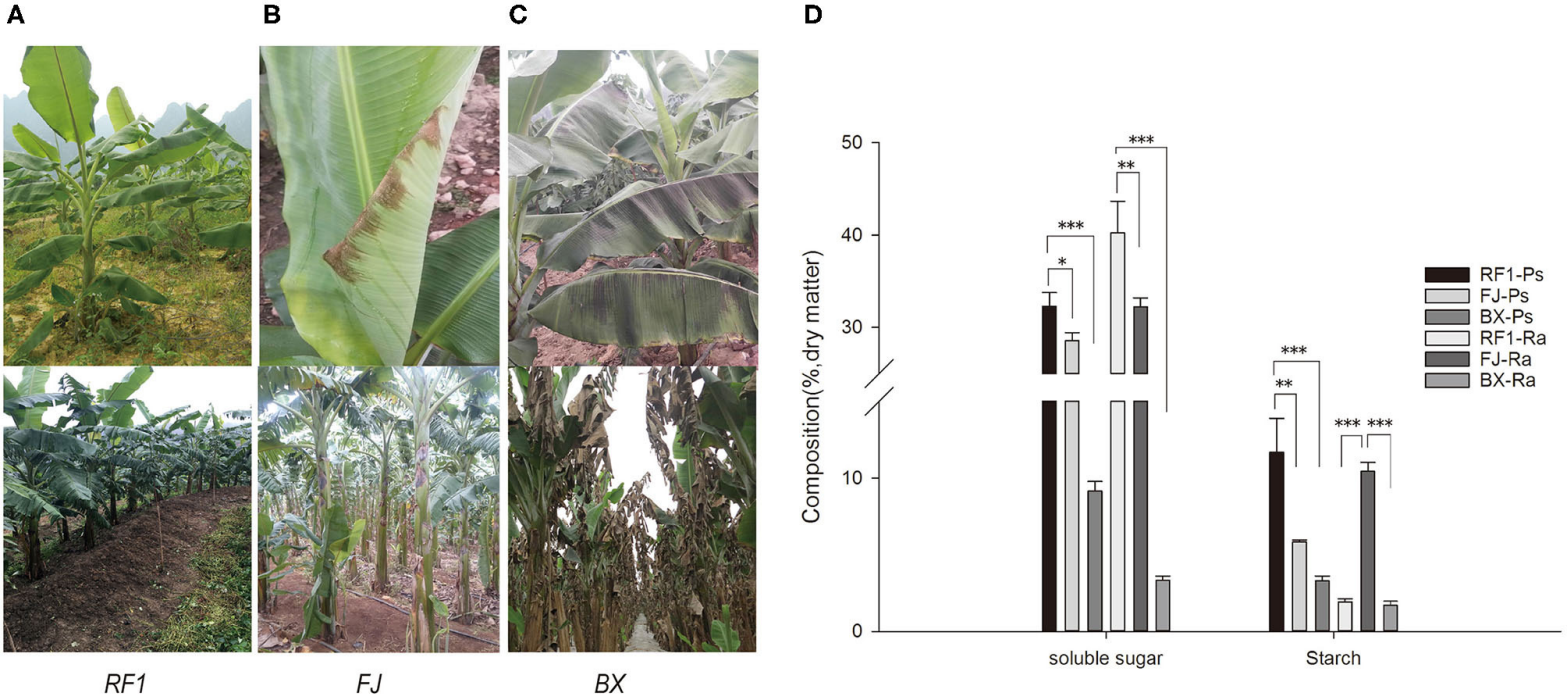
Phenotype observations of cold tolerance and assessment of carbohydrate accumulation in *RF1* mutant and cultivars (FJ and BX). **(A–C)** Leaf phenotypes during growth in winter at the Xingyi test site. The top images of the leaf were taken after 3 days of chilling injury. The bottom images of the leaf were taken after 14 days of chilling injury. **(D)** Contents of soluble sugar and starch in the pseudo-stem and rachis. Data are the mean ± SD (*n* = 3); One-way ANOVA test was used to assess statistical significance (*p < 0.05; **p < 0.01; ***p < 0.001).

Banana is suitable for planting in tropical and subtropical regions, as it is highly susceptible to chilling injury. Accordingly, this study investigated the tolerance of *RF1* to cold conditions. In the winter of 2014, at the Xingyi plot (Guizhou Province) there was a sudden drop in temperatures that reached as low as 1°C for 3 d, during which time we observed that the *RF1* blade did not have any cold injury syndromes, producing normal flower buds (Figure 4A). In contrast, the FJ cultivar exhibited mild injury symptoms in its blade, and another cultivar Baxijiao displayed severe chilling symptoms in the whole plant, leading to death (Figures 4B,C).

Low temperatures usually induce extracellular ice formation, which results in cell deformation and chilling damage in plants (Takahashi et al., 2021). The contents of soluble sugar, proline, soluble protein, and the cell wall composition will together strongly influence the extent and magnitude of cell deformation induced by chilling damage (Pearce, 1988; Xu et al., 2010; Takahashi et al., 2021). Therefore, to understand why the *RF1* mutant was capable of high cold resistance, we examined its cell wall composition and other components of pseudo-stem and rachis tissues (Table 4). Compared with the two control cultivars (FJ and BX), the *RF1* mutant contained 2–8 times higher levels of soluble sugars and starch in both its pseudo-stem and rachis tissues (Figure 4D), which could be a major factor explaining its enhanced cold resistance. Notably, the BX cultivar contained the lowest levels of soluble sugars and starch, accounting for its most injury of the whole plant. Further, in the pseudo-stem tissues, the cellulose level of the *RF1* mutant was relatively reduced vis-à-vis the FJ and BX cultivars. Taken together, these results indicated that the carbon partitioning processes were likely altered in the *RF1* mutant via regulation of starch–sucrose metabolisms.

**Table 4:** Cell wall composition (% dry matter) of pseudo-stem and rachis tissues in the *RF1* and FJ samples.

**Samples**	**Soluble sugar**	**Starch**	**Pectin**	**Cellulose**	**Hemicellulose**	**Lignin**	**Ash**
RF1-Ps#	34.3 ± 1.600b	15.0 ± 0.610a	5.7 ± 0.203a	27.5 ± 0.499c	12.2 ± 0.420b	7.9 ± 0.548e	1.4 ± 0.0642d
FJ-Ps	28.7 ± 0.696d	5.8 ± 0.239c	5.9 ± 0.247a	35.5 ± 0.694b	11.9 ± 0.689b	10.8 ± 0.698c	1.7 ± 0.125c
BX-Ps	10.6 ± 0.624e	4.4 ± 0.501d	5.1 ± 0.462ab	38.7 ± 2.115a	15.1 ± 0.266a	12.6 ± 0.317b	2.1 ± 0.149b
RF1-Ra^*^	45.4 ± 0.380a	2.4 ± 0.36e	3.8 ± 0.427b	24.1 ± 1.700d	10.4 ± 0.138c	9.3 ± 0.884d	3.5 ± 0.164a
FJ-Ra	31.3 ± 0.336c	10.2 ± 0.413b	4.1 ± 0.224b	28.4 ± 1.423c	10.6 ± 0.518c	11.5 ± 0.319c	3.4 ± 0.110ab
BX-Ra	5.03 ± 0.446f	2.8 ± 0.275e	3.9 ± 0.425b	21.2 ± 2.240e	12.8 ± 0.352b	15.5 ± 0.240a	3.7 ± 0.103a
95% LSD	2.902	1.695	0.620	2.826	0.773	0.982	0.220

# *Ps, pseudo-stem;*

* *Ra, rachis*.

*Data are the mean ± SD (n = 10). Statistical significance was assessed using one-way ANOVAs. Within columns, differing lowercase letters indicate group values that are significantly different (one-way ANOVA, p < 0.05)*.

Sigatoka leaf spot disease, caused by *Mycosphaerella fijiensis*, has become the most damaging disease impacting banana crops worldwide (Vishnevetsky et al., 2011). The development of banana varieties with Sigatoka disease resistance is currently the dominant strategy for improving tolerance to Sigatoka. Accordingly, this study also evaluated the resistance of *RF1* to Sigatoka disease in different experimental fields (Table 5). Field observations of 2-year experiments showed that the *RF1* mutant has significantly improved quantitative traits, such as YLS, NSL, and INSL at *p* < 0.05, when compared to the FJ cultivar (Table 5). These results indicated the *RF1* mutant harbors relatively high resistance to Sigatoka disease, which is a major biotic problem limiting banana growth and development.

**Table 5:** Comparison of banana resistance to Sigatoka disease in *RF1* and FJ grown at two experimental filed sites.

**Test site**	**Harvest year**	**RF1**			**FJ**		
		**YSL**	**NSL**	**INSL**	**YSL**	**NSL**	**INSL**
Haikou	FS	3.1 ± 0.316a	10.2 ± 0.422a	20.6 ± 3.376a	3.8 ± 0.422b	8.8 ± 0.422b	31.9 ± 5.399b
	FR	3.4 ± 0.516a	8.8 ± 0.632a	27.3 ± 5.753a	4.1 ± 0.568b	7.8 ± 0.422b	40.0 ± 8.593b
Xingyi	FS	2.7 ± 0.675a	10.0 ± 0.943a	17.1 ± 6.815a	2.8 ± 0.632b	8.9 ± 0.738b	27.1 ± 6.008b
	FR	3.4 ± 0.516a	8.9 ± 0.738a	20.3 ± 7.081a	3.5 ± 0.527b	7.6 ± 0.516b	33.2 ± 8.213b

*YLS, youngest leaf with at least 10 necrotic spots at flowering (youngest leaf spotted); NSL, number of standing leaves at flowering (NSL); INSL, the index of non-spotted leaves*.

*Data are the mean ± SD (n = 10). Statistical significance was assessed using one-way ANOVAs. Within columns, differing lowercase letters indicate group values that are significantly different (one-way ANOVA, p < 0.05)*.

## Discussion

Traditional genetic breeding has struggled to improve major agronomic traits and stress resistance in bananas, mainly due to its parthenocarpy and polyploidy (Ortiz and Swennen, 2014; Amah et al., 2019). Therefore, mutation breeding using *in vitro* propagated bananas has been proposed as a powerful approach to develop new cultivars or improved strains based on excellent cultivars (Karmarkar et al., 2001; Saraswathi et al., 2016; Datta et al., 2018). The mutagenic frequency of progenies can be increased to ca. 11%, a level 100 times that of the natural (background) mutagenic frequency (Karmarkar et al., 2001). In this study, we used chemical mutagens to treat adventitious buds in banana plants, which can greatly increase the overall mutagenic efficiency. Our study was able to generate a wide range of mutation types, well-beyond that which occurs under natural variation.

Research shows that when using mutagens in a high concentration, both the toxicity and physiological damage to recipient plants are relatively increased, which often affects their rate of survival (Gao et al., 2018; Samatadze et al., 2019). Conversely, when treating plants with a low concentration of mutagen for a long time at low temperature, the damage to chemicals in cells is limited, because the low temperature confers to chemicals certain stability, and the low concentration has little or negligible adverse impacts upon cells, which jointly can improve the survival rate and mutagenic efficiency. Provided that banana is used for the aseptic proliferation of adventitious buds, even at a lower concentration, it could produce a higher effect because soaking can make the cell metabolism of adventitious buds active and sensitive to the applied mutagens. Here we found that 0.8% v/v EMS for 4 h was the optimal situation for inducing a high mutation frequency in the FJ shoot tips. We describe here the identification of a stably inherited mutant ‘*Refen 1*’ (*RF1*) with a semi-dwarfing phenotype and significantly better agronomic traits than the wild type (local cultivars).

Dwarfism is one of the most important objectives of banana breeding because dwarf plants have strong resistance to wind or flood disturbances (Cho et al., 2016; Wang et al., 2021). Recent work found that chemical mutagenesis is capable of inducing dwarf and semi-dwarf mutants from high-stem banana varieties (Aslam et al., 2016; Amosova et al., 2019). In our study, the gene locus controlling banana plant height was sensitive to the EMS mutagen, leading to abundant dwarf or semi-dwarf mutant offspring obtained. Hence, our approach provides a novel avenue for the semi-dwarf breeding of new banana varieties, yet the molecular mechanisms responsible for the dwarfism induced by EMS still await elucidation in future studies.

Excellent fruit quality is another key goal of banana breeding, and primary carbohydrate metabolites are essential determinants of fruit quality (Allegra et al., 2018). It is known that mutagens can induce further variation in quality traits, such as astringency, this being relatively easy to remove via mutagenesis (Shen et al., 2019). Banana is a typical climacteric fruit, one whose fruit-flavor quality is affected by a series of ripening processes that progress in tandem with the accumulation of soluble sugars. Sugar is the ultimate precursor for most quality-relevant components in the fruit of bananas, such as acids, pigments, tannins, and aroma volatiles (Hall et al., 2011). A high acid content often reduces fruit quality, though a moderate concentration of acid can improve the palatability of fruits (Liao et al., 2019). Therefore, the quality of a fruit and its taste are usually influenced by the sugar and acid contents or, more simply, by its sugar-to-acid ratio (Qiao et al., 2018). In our study, the EMS-induced mutant gained a RSTA in an efficient manner, in contrast to the long timespan needed to achieve that through traditional breeding.

Plant cell walls have critical biological roles in plant growth and development, such as the regulation of cell shape and expansion, ion exchange, and resistance to biotic or abiotic stresses. Recently, carbon partitioning was also characterized to function in regulating the biosynthesis of cell wall polysaccharides and the production of other carbohydrates, such as soluble sugars and starch (Fan et al., 2017, 2019). That the *RF1* mutant harbors remarkably augmented levels of soluble sugars and starch accumulation, coupled with its significantly reduced cellulose deposition, strongly suggests that dynamic regulation of carbon partitioning is operating in this discovered banana mutant. Correspondingly, the considerable soluble sugar accumulation in the *RF1* mutant is likely a major cause of its high resistance to cold stress, whereas the altered cell wall composition would contribute to its dwarf phenotype (Corneillie et al., 2019; Touchell et al., 2020; Madadi et al., 2021a,b).

## Conclusion

Using classic EMS-induced mutagenesis, this study identified a novel banana *RF1* mutant that has a typical semi-dwarf phenotype. Compared with local banana cultivars, the *RF1* mutant showed significantly improved agronomic traits and enhanced tolerance to cold stress and resistance to Sigatoka disease. Chemical analyses further indicated that dynamic regulation of carbon partitioning should occur in the *RF1* mutant to explain its remarkably augmented soluble sugars and starch accumulation and reduced cellulose deposition. Therefore, this study has not only demonstrated a genetic approach for selecting desirable bananas, but also enhanced our understanding of the genes controlling plant architecture, tolerance to cold and resistance to Sigatoka disease, and carbohydrate metabolism in bananas. Further work is now needed to examine the molecular mechanisms underlying the improved traits of the *RF1* mutant to develop genetically improved banana cultivars for widespread use.

## Data Availability Statement

The original contributions presented in the study are included in the article/Supplementary Material, further inquiries can be directed to the corresponding author.

## Author Contributions

XW, AW, YL, and JL conceived and designed the work. AW, YL, YX, QW, JW, FLin, DG, FLiu, and YW performed the experiments and carried out the analyses. XW, LP, and JL wrote the manuscript. All authors contributed to the article and approved the submitted version.

## Funding

This work was financially supported by the Youth Foundation of Natural Science Foundation of Hainan Province (320QN306), the China Agriculture Research System of MOF and MARA (CARS-31-02), and the Lancang-Mekong Cooperation Special Fund.

## Conflict of Interest

The authors declare that the research was conducted in the absence of any commercial or financial relationships that could be construed as a potential conflict of interest.

## Publisher's Note

All claims expressed in this article are solely those of the authors and do not necessarily represent those of their affiliated organizations, or those of the publisher, the editors and the reviewers. Any product that may be evaluated in this article, or claim that may be made by its manufacturer, is not guaranteed or endorsed by the publisher.
