# Corrigendum: A Novel Banana Mutant “*RF 1*” (*Musa* spp. ABB, Pisang Awak Subgroup) for Improved Agronomic Traits and Enhanced Cold Tolerance and Disease Resistance

**DOI:** 10.3389/fpls.2021.796904

**Published:** 2021-11-08

**Authors:** Xiaoyi Wang, Anbang Wang, Yujia Li, Yi Xu, Qing Wei, Jiashui Wang, Fei Lin, Deyong Gong, Fei Liu, Yanting Wang, Liangcai Peng, Jingyang Li

**Affiliations:** ^1^Hainan Banana Healthy Seedling Propagation Engineering Research Center, Haikou Experimental Station, Chinese Academy of Tropical Agricultural Sciences, Haikou, China; ^2^The Fruit Tree Research Center, Institute of Subtropical Crops, Guizhou Academy of Agricultural Sciences, Xinyi, China; ^3^Biomass and Bioenergy Research Centre, College of Plant Science and Technology, Huazhong Agricultural University, Wuhan, China

**Keywords:** “*ReFen 1*” mutant, Pisang Awak (ABB), ethyl methanesulfonate (EMS)-mutagenesis, semi-dwarfing, agronomic traits, cold tolerance, sigatoka disease resistance, banana breeding

In the published article, there was an error regarding the order of funding agencies as listed in the Funding statement. The correct Funding statement appears below.

## Funding

This work was financially supported by the Youth Foundation of Natural Science Foundation of Hainan Province (320QN306), the China Agriculture Research System of MOF and MARA (CARS-31-02), and the Lancang-Mekong Cooperation Special Fund.

The authors apologize for this error and state that this does not change the scientific conclusions of the article in any way. The original article has been updated.

## Publisher's Note

All claims expressed in this article are solely those of the authors and do not necessarily represent those of their affiliated organizations, or those of the publisher, the editors and the reviewers. Any product that may be evaluated in this article, or claim that may be made by its manufacturer, is not guaranteed or endorsed by the publisher.

