# Supplementary material for: A Novel Banana Mutant “RF 1” (Musa spp. ABB, Pisang Awak Subgroup) for Improved Agronomic Traits and Enhanced Cold Tolerance and Disease Resistance
Source: Front Plant Sci. 2021 Sep 23;12:730718. doi: 10.3389/fpls.2021.730718 (PMC8496975; doi:10.3389/fpls.2021.730718)
Supplement: Supplementary file 1 [file Data_Sheet_1.docx]

**
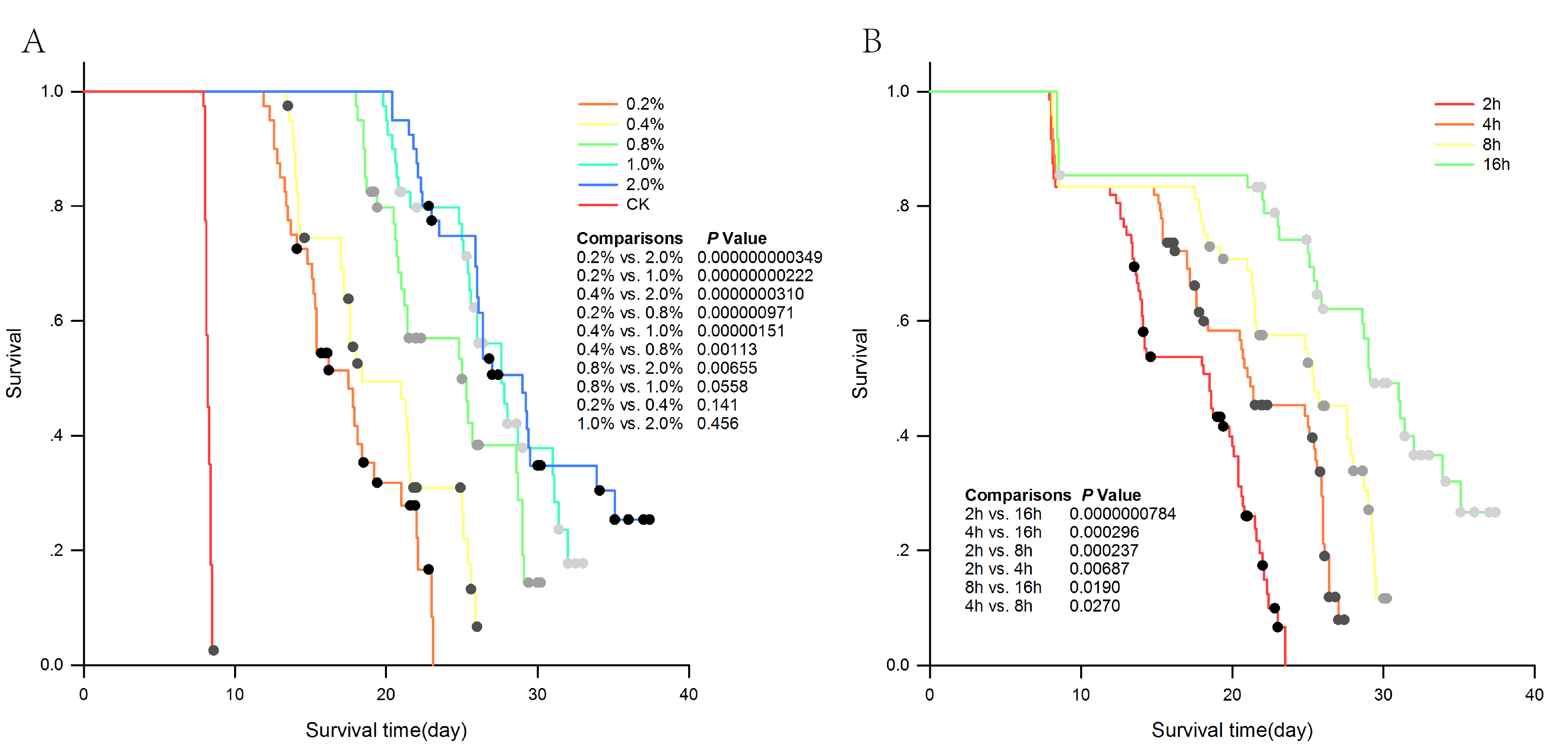
**

**Supplementary Figure 1.** Gehan-Breslow survival curves of banana multi-shoots growth and death during the EMS mutagenesis. Each treatment included 60 shoots , and the Gehan–Breslow test performed as a statistically significant difference between survival curves in various EMS concentration (A) and treat time (B).


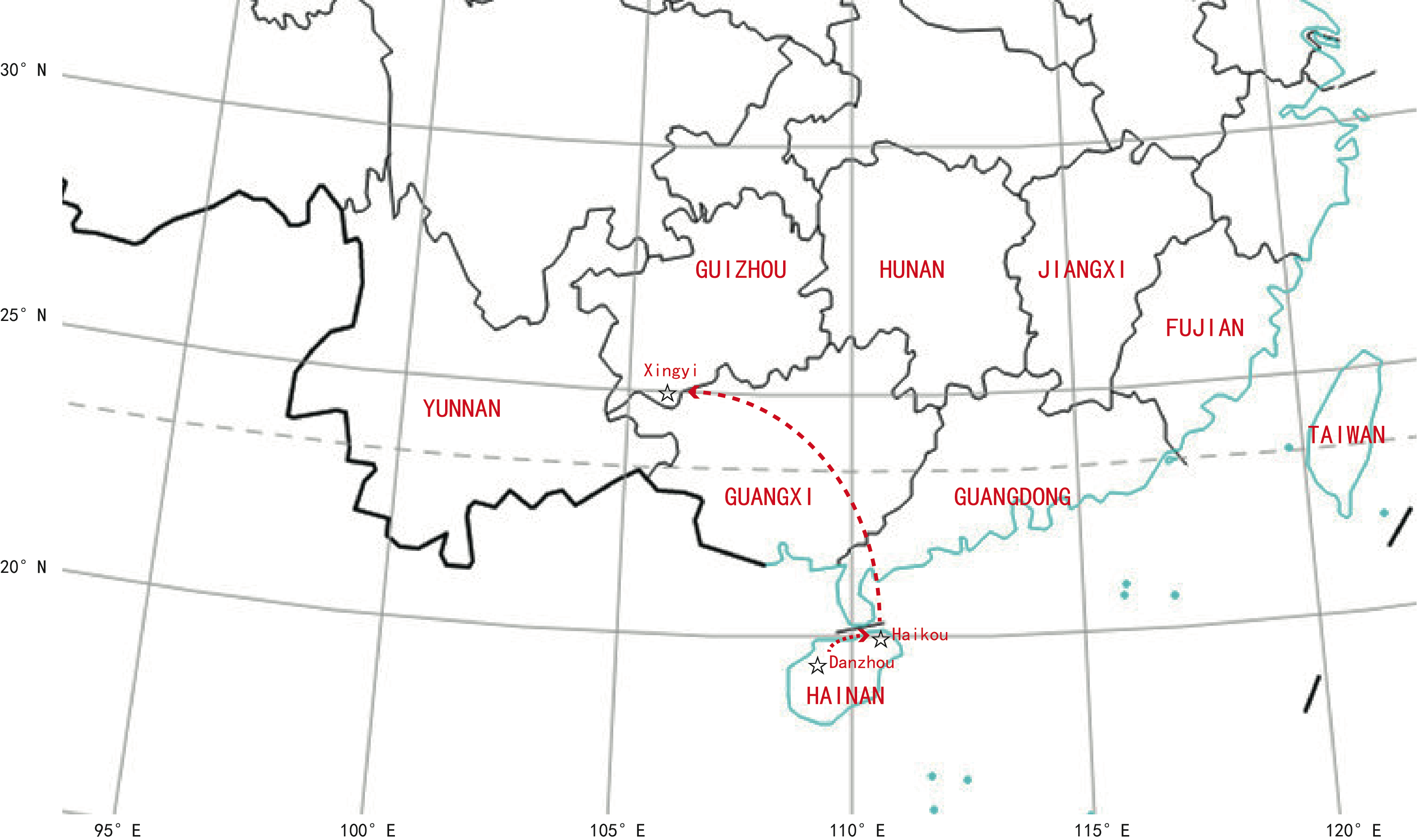


**Supplementary Figure 2.** A map showing three experimental locations for banana growth (☆represent test site).

**Supplementary Table 1. Information of experimental field conditions for growing *RF1* mutant.**

| **Location name of site** | **Temperature*** | | **Annual average rainfall**** | **Evaluation date** | **Assessment** |
| --- | --- | --- | --- | --- | --- |
|  | **AMMIT** | **AMMAT** |  |  |  |
| **Danzhou, Hainan province.**  **(altitude: 130m)** | **20.5℃** | **28.8℃** | **1856.5 mm** | **Planted in May 2014, successive two ratoons cultivation.** | **Agronomic characteristics, Plant and ratoon crop harvest** |
| **Haikou, Hainan province.**  **(altitude: 34m)** | **22.2℃** | **28.5℃** | **1645.5 mm** | **Planted in June 2014, successive two ratoons cultivation.** | **Black sigatoka, new plant and ratoon plant.** |
| **Xingyi, Guizhou province.**  **(altitude: 1138m)** | **13.3℃** | **21.1℃** | **1451 mm** | **Planted in March 2015, one ratoon cultivation.** | **Cold resistance, Plant crop at bunching** |

AMMIT, annual mean minimum temperature;

AMMAT, annual mean maximum temperature;

Note: *, **：The climatic data were sourced from the National Climatic Data Center （http://data.cma.cn/site/index.html）.
